# Platelet‐to‐Neutrophil Ratio: A Novel Prognostic Indicator for Anti‐PD‐1‐Based Therapy in Relapsed/Refractory Hodgkin Lymphoma and Solid Tumors

**DOI:** 10.1002/mco2.70199

**Published:** 2025-05-16

**Authors:** Yuting Pan, Xin Zhang, Chunmeng Wang, Nannan Lu, Yang Liu, Yixin Chang, Xueting Qin, Weidong Han, Jing Nie

**Affiliations:** ^1^ Department of Bio‐therapeutic, The First Medical Centre Chinese People's Liberation Army General Hospital Beijing China; ^2^ Medical School of Chinese People's Liberation Army Beijing China; ^3^ Changping Laboratory Beijing China

**Keywords:** peripheral biomarker; platelet‐to‐neutrophil ratio, prognosis, Hodgkin lymphoma, anti‐PD‐1 immunotherapy

## Abstract

Program cell death‐1 (PD‐1) blockade treatment has been shown effective in cases with relapsed/refractory classical Hodgkin Lymphoma (R/R cHL), while prognostic biomarkers remain unclear. Seventy‐seven cases with R/R cHL who received immunotherapy for the first time were included. Receiver operator characteristic analysis displayed platelet‐to‐neutrophil ratio (PNR) as the most probable indicator among distinct inflammatory‐cell ratios. Patients with high pretreatment PNR (≥ 51.6) achieved significantly higher complete response (CR) rate as compared with patients with low PNR (< 51.6), and PNR^high^ patients displayed significantly longer progression‐free survival (PFS) versus PNR^low^ patients (*p* = 0.001). Cox analysis indicated PNR as an independent factor for prognosis (hazard ratio, 0.34, 95% CI, 0.18–0.65, *p* = 0.001). Among patients acquiring CR, higher PNR was associated with improved PFS and relapse‐free survival. Moreover, PNR correlations with CR rate and PFS were validated in external cohort of cHL. Notably, PNR was also a strong prognostic biomarker for PFS and overall survival after anti‐PD‐1 combination therapy in patients with solid tumors, such as biliary tract carcinoma, gastric carcinoma, or colon cancer. In conclusion, this study for the first time reveals a correlation between pretreatment peripheral PNR and prognosis of anti‐PD‐1‐based therapy in patients with relapsed/refractory cHL and advanced solid tumor.

## Graphical abstract

Patients with relapsed/refractory cHL who had high pretreatment peripheral platelet‐to‐neutrophil ratio acquired increased complete response rate and longer progression‐free survival after anti‐PD‐1‐based therapy. Additionally, the prognostic value of platelet‐to‐neutrophil ratio was validated in other cohorts of patients with advanced solid tumors, such as biliary tract carcinoma, gastric carcinoma, colon cancer, and melanoma, as well as an external relapsed/refractory cHL cohort.

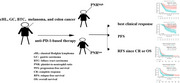

## 1 Introduction

Hodgkin lymphoma (HL) belongs to an uncommon B‐cell lymphoid malignancy, affecting approximately 8570 new cases each year in the United States [1]. The onset age of HL exhibits a bimodal distribution, with the highest incidence in youngsters and persons aged 60 and above [2, 3]. The classical Hodgkin lymphoma (cHL) accounts for 95% of HL and characterized by a unique tumor microenvironment containing only a few malignant tumor cells but abundant inflammatory immune cells. The occurrence of HL might originate from Epstein–Barr virus (EBV) infection, abnormal activation of NF‐κB, and impaired transcription factor networks [4].

HL is a highly curable disease diagnosis at early stages. For cases with relapsed/refractory cHL (R/R cHL), the NCCN Guidelines recommend second‐line systemic therapy regimens, such as brentuximab vedotin (BV), programmed cell death 1 (PD‐1) blockade therapy, chemotherapy, and their combinations, or radiotherapy, followed by autologous stem cell transplantation (ASCT) [5]. Among cases with R/R cHL who were transplant‐ineligible or after ASCT therapy, anti‐PD‐1 monotherapy showed a high objective response (65%–87%) [6, 7, 8]. To maximize the complete response (CR) rate, we previously reported that among the anti‐PD‐1 naive patients, the synergistic use of DNA demethylating agent decitabine and anti‐PD‐1 camrelizumab showed a markedly higher CR rate (79% vs. 32%) and improved PFS (35 months vs. 20 months) as compared with camrelizumab alone [9]. However, a subset of cases had primary resistance, or experienced disease progression or relapse after the initial response.

Currently, the traditional approach to assess whether cancer patients benefit from anti‐PD‐1 includes the detection of programmed cell death ligand 1 (PD‐L1) expression, microsatellite instability, and tumor mutation burden (TMB) from tumor tissues [10, 11, 12]. These conventional biomarkers for forecasting the efficacy of immunotherapy, although holding some clinical application value, also display notable limitations. Take TMB for example, first, the thresholds vary across different studies, and a unified standard is lacking; second, the predictive power of TMB is influenced by cancer types, exhibiting variable efficacy in different malignancies; furthermore, although TMB can indicate the potential of tumors to produce neoantigens, it is unable to comprehensively reflect the status of the immune microenvironment; lastly, the detection of TMB necessitates high‐throughput sequencing, which entails substantial costs [13].

With the advancement of immunotherapy, researchers have increasingly focused attention on the peripheral blood biomarkers to predict response of immunotherapy. Peripheral blood samples are readily obtainable, minimally invasive to collect, and amenable to repeated sampling, rendering them ideal for the dynamic monitoring of treatment outcomes. Previous studies have shown that neutrophil‐to‐lymphocyte ratio (NLR) and platelet‐to‐lymphocyte ratio (PLR) were associated with the efficacy and survival of tumor patients following anti‐PD‐1 therapy [14, 15]. Higher baseline NLR and PLR typically correlate with inferior immunotherapy outcomes. The increase in NLR and PLR may mechanistically indicate various pathophysiological alterations within the body, notably inflammatory reactions and immunosuppressive conditions, along with their intricate interactions with the tumor microenvironment [16].

However, whether the ratios of peripheral immune‐inflammatory cells could serve as predictive biomarkers in cHL with anti‐PD‐1‐based immunotherapy is still unclear. In this research, we reported the prognostic value of a novel peripheral indicator “platelet‐to‐neutrophil ratio (PNR)” in cases with R/R cHL after treated with anti‐PD‐1‐based immunotherapy and further validated in other solid tumor patients.

## 2 Results

### 2.1 Baseline Characteristics of Patients With cHL

During 2017–2019, a total of 77 cases with R/R cHL who received anti‐PD‐1 camrelizumab monotherapy (*n* = 26) or decitabine‐plus‐camrelizumab combination (*n* = 51) for the first time were included in this study. Overall, median age was 27 years, 71% of patients received three or more previous lines, 20 cases (26%) underwent ASCT and others were transplant‐ineligible, and none were treated with anti‐PD‐1/PD‐L1 immunotherapy before. At data cutoff on April 1, 2024, 74 patients (96%) acquired objective responses (46 for CR, 28 for PR), and 56 patients experienced disease progression either on treatment (*n* = 55) or after treatment discontinuation (*n* = 1). An overview clinical characteristics of all cases is shown in Table 1.

**TABLE 1:** Clinicopathological variables of patients with R/R cHL.

Characteristics	Total (*n* = 77)	PNR^high^ subgroup (*n* = 28)	PNR^low^ subgroup (*n* = 49)	*p* value
Age				0.259
< 27	34 (44.2)	10 (35.7)	24 (49)	
≥ 27	43 (55.8)	18 (64.3)	25 (51)	
Sex				0.760
Female	32 (41.6)	11 (39.3)	21 (42.9)	
Male	45 (58.4)	17 (60.7)	28 (57.1)	
Histology				0.477
NSHL	48 (62.3)	16 (57.1)	32 (65.3)	
Non‐NSHL	29 (37.7)	12 (42.9)	17 (34.7)	
Stage				0.895
II—III	31 (40.3)	11 (39.3)	20 (40.8)	
IV	46 (59.7)	17 (60.7)	29 (59.2)	
Previous ASCT				0.219
Yes	20 (26)	5 (17.9)	15 (30.6)	
No	57 (74)	23 (82.1)	34 (69.4)	
Lines of prior therapy				0.294
< 3	22 (28.6)	10 (35.7)	12 (24.5)	
≥ 3	55 (71.4)	18 (64.3)	37 (75.5)	
Immunotherapy regimen				0.466
Camrelizumab monotherapy	26 (33.8)	8 (28.6)	18 (36.7)	
Decitabine‐plus‐camrelizumab	51 (66.2)	20 (71.4)	31 (31.5)	

*Note*: Data are presented as no. (%). A *p* < 0.05 is considered to indicate statistical significance. The *p* values are calculated in SPSS 26.0 using *χ* ^2^ test.

Abbreviations: ASCT, autologous stem cell transplant; NSHL, nodular sclerosis Hodgkin lymphoma; PNR, platelet‐to‐neutrophil ratio.

For the purpose of external validation, an external cohort consisting of 50 cases with R/R cHL who underwent anti‐PD‐1‐based immunotherapy for the first time during 2016–2024 at Department of Hematology in the Fifth Medical Center of the Chinese People's Liberation Army General Hospital was used. In this cohort, median age was 29 years, 22% of patients were treated with decitabine‐plus‐anti‐PD‐1 therapy, and others received chemotherapy‐plus‐anti‐PD‐1 treatment. At data cutoff on January 26, 2025, 41 patients (82%) acquired objective responses (18 for CR and 23 for PR) and 32 patients experienced disease progression. An overview clinical feature of external validation cases is displayed in Table S1.

### 2.2 ROC Analysis and Grouping in R/R cHL

**FIGURE 1:**
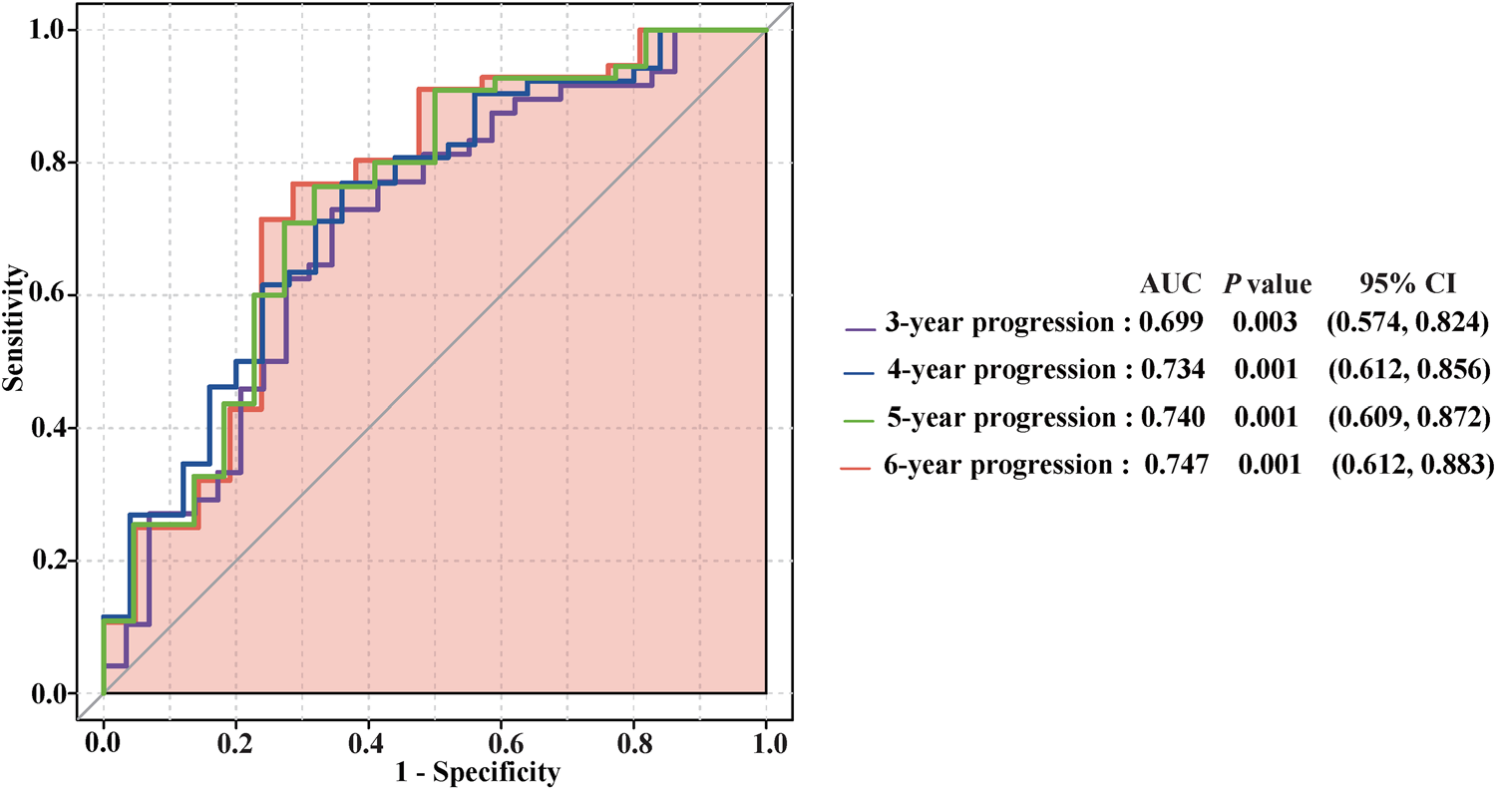
ROC of PNR in R/R cHL. ROC curves of pretreatment PNR level to assess tumor progression at the indicated years in patients with R/R cHL after anti‐PD‐1‐based therapy. PNR curves for 3‐year, 4‐year, 5‐year, and 6‐year progression are indicated as purple line, blue line, green line, and orange line, respectively, and gray line indicates reference line. AUC, area under ROC curve; CI, confidence interval; PNR, platelet‐to‐neutrophil ratio; ROC, receiver operator characteristic.

The receiver operating characteristic (ROC) curves were plotted to screen baseline peripheral blood composite biomarkers that could predict disease progression on immunotherapy for R/R cHL patients and to calculate the optimal cut‐off values. Among different ratios of the immune‐inflammatory cells, such as LMR, PLR, NLR, PNR as well as platelet and neutrophil counts, PNR served as the strongest prognostic biomarker (Figure S1). PNR had prominent value in predicting disease progression at multiple years, and the sensitivity and specificity for 6‐year progression were 71.4% and 76.8%, respectively, with the area under ROC curve (AUC) being 0.747 (Figure 1). According to its optimal cut‐off value of 51.6, patients were divided into PNR^high^ (PNR ≥ 51.6, *n* = 28) and PNR^low^ (PNR < 51.6, *n* = 49) subgroups.

Chi‐square test was used to analyze the relationship between clinicopathological characteristics and PNR, as indicated by age, sex, histology, number of lines of prior therapy, and immunotherapy regimen. As shown in Table 1 and Table S1, there was no significant difference between the two subgroups.

### 2.3 Association of PNR and Best Clinical Response of Immunotherapy in R/R cHL

Numerous reports have reported that patients with R/R cHL achieved high clinical response after anti‐PD‐1‐containing therapy, especially for cases who did not have previous anti‐PD‐1 treatment. Among the entire 77 patients in this study, 46 (60%) patients achieved CR, 28 evaluated as partial response, and three had stable disease or progressive disease. Remarkably high objective response rates were detected in these two subgroups, while cases in the PNR^high^ subgroup were more likely to achieve CR as compared with cases in the PNR^low^ subgroup (75% vs. 51%, *p* = 0.039; Table 2). Strikingly, patients with high PNR level obtained increased CR rate versus those with low PNR level in patients who had previous ASCT (*p* = 0.035). Additionally, among the transplant‐ineligible patients, or patients with different prior treatment lines (< 3 or ≥ 3), higher CR rates were also observed in the PNR^high^ subgroup versus those in the PNR^low^ subgroup, although the statistical significance were not reached (Table 2). Consistently, in the external validation cohort with R/R cHL, we also observed that cases in the PNR^high^ subgroup acquired higher CR rate as compared with those in the PNR^low^ subgroup (57% vs. 21%, *p* = 0.008; Table S2).

**TABLE 2:** Association of PNR and the best clinical response of anti‐PD‐1‐based therapy in patients with R/R cHL.

Best clinical response	Overall	PNR^high^ subgroup	PNR^low^ subgroup	*p* value
**Objective response rate**				
All patients	74 (96%)	26 (93%)	48 (98%)	0.266
**CR rate**				
All patients	46 (60%)	21 (75%)	25 (51%)	0.039
Patients with prior ASCT	12 (60%)	5 (100%)	7 (47%)	0.035
Patients ineligible for ASCT	34 (60%)	16 (70%)	18 (53%)	0.209
Patients with < 3 prior lines	16 (73%)	9 (90%)	7 (58%)	0.097
Patients with ≥ 3 prior lines	30 (55%)	12 (67%)	18 (49%)	0.208

*Note*: Data are presented as no. (%). A *p* < 0.05 is considered to indicate statistical significance. The *p* values are calculated in SPSS 26.0 using *χ* ^2^ test.

### 2.4 Association of PNR and Progression‐free Survival in R/R cHL

**FIGURE 2:**
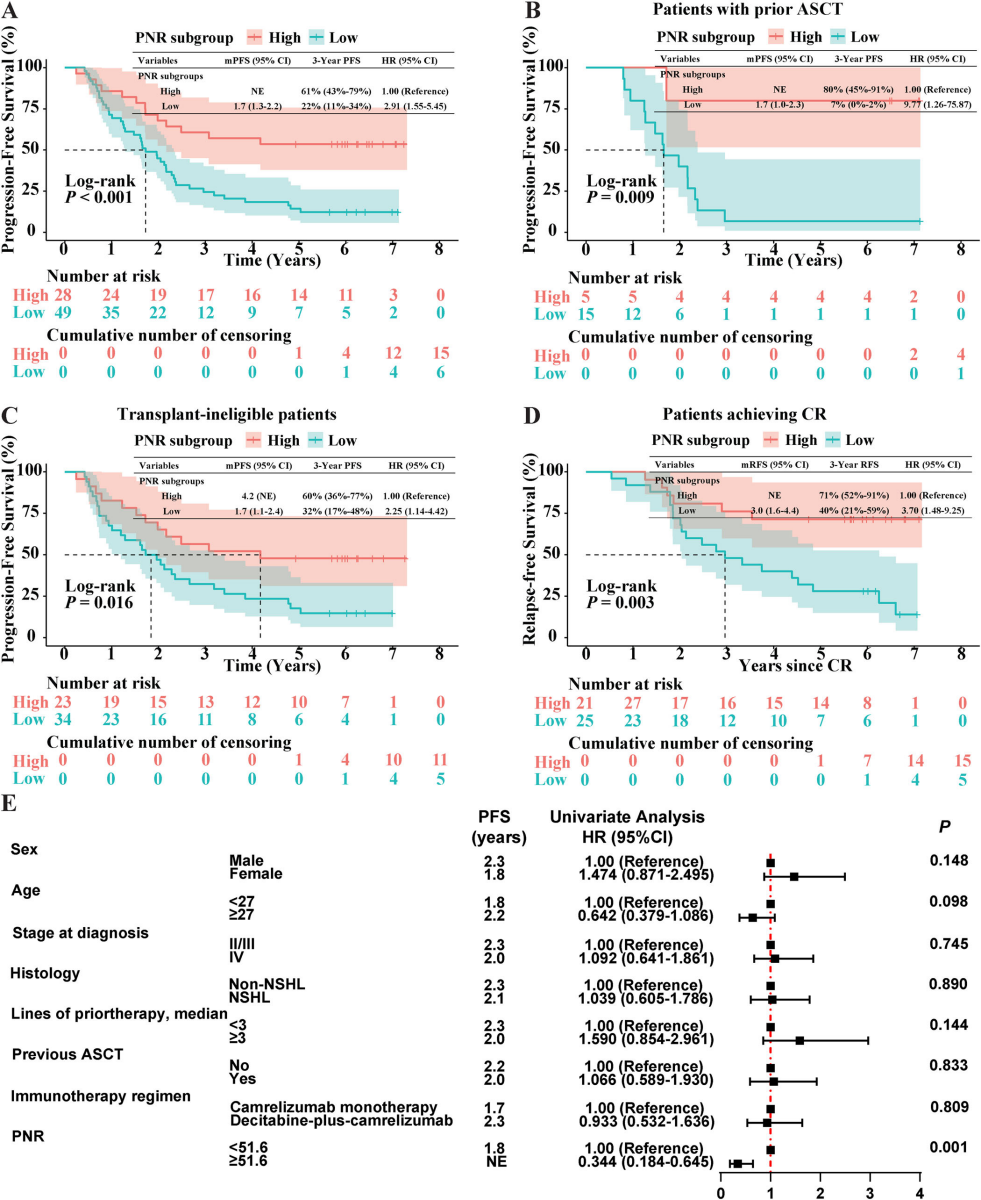
Prognostic value of PNR in patients with R/R cHL after receiving anti‐PD‐1‐based therapy. (A) Kaplan–Meier survival curves of progression‐free survival (PFS) in patients with high and low PNR levels. Median PFS (95% CI) and 3‐year PFS rate (95% CI) are shown. Plus signs indicate censored data. (B and C) Kaplan–Meier survival curves of PFS in patients who had previous ASCT (B) or those ineligible for transplantation (C) with high and low PNR levels. Median PFS (95% CI) and 3‐year PFS rate (95% CI) are shown. Plus signs indicate censored data. (D) Kaplan–Meier survival curves of relapse‐free survival (RFS) in patients achieving CR with high and low PNR levels. Median RFS (95% CI) and 3‐year RFS rate (95% CI) are shown. Plus signs indicate censored data. (E) Forest plots showing the result of univariate Cox regression on PFS. NE, not evaluated.

By data cutoff date of April 1, 2024, after a median follow‐up of 6.0 years (range, 4.7–7.2), 56 patients had disease progression. The overall median progression‐free survival (PFS) was 2.2 years (95% confidence interval [CI], 1.8–2.5). We next compared PFS within distinct PNR subgroups. Consistently with a higher CR rate, median PFS was not reached in the PNR^high^ subgroup, as compared with 1.7 years (95% CI, 1.3–2.2) in the PNR^low^ subgroup (*p* < 0.001; Figure 2A). PFS rate at 3 years was 61% (95% CI, 43%–79%) in PNR^high^ subgroup versus 22% (95% CI, 11%–34%) in PNR^low^ subgroup (Figure 2A). For patients who previously treated with ASCT (*n* = 20) or those ineligible for transplantation (*n* = 57), higher pretreatment PNR level also associated with improved PFS (*p* = 0.009 and 0.016, respectively; Figure 2B,C). Moreover, cases with high PNR level had longer PFS versus those with low PNR level among patients whose previous treatment lines either ≥ 3 or < 3 (*p* = 0.021 and 0.016, respectively; Figure S2A,B).

Among the 46 patients who obtained CR following anti‐PD‐1‐based therapy, median PFS was not reached in PNR^high^ patients versus 2.7 years (95% CI, 1.7–3.7) in PNR^low^ patients (*p* = 0.002; Figure S2C). Strikingly, patients in the PNR^high^ subgroup achieved significantly superior relapse‐free survival (RFS) versus that in PNR^low^ subgroup (*p* = 0.003), with 3‐year RFS rates of 71% (95% CI, 52%–91%) and 40% (95% CI, 21%–59%) in PNR^high^ and PNR^low^ patients, respectively (Figure 2D).

The univariate regression analysis elucidated, the risk of disease progression after anti‐PD‐1‐containing treatment in the PNR^high^ subgroup was obviously lower than that in the PNR^low^ subgroup (HR = 0.34; 95% CI, 0.18–0.65; *p* = 0.001; Figure 2E). After adjusting for number of lines of prior therapy, sex, age, and immunotherapy regimen, PNR was found to have significant association with PFS in R/R cHL patients treated with anti‐PD‐1‐based therapy (Figure 2E).

### 2.5 Association of PNR With the Efficacy of Different Anti‐PD‐1 Regimens

**FIGURE 3:**
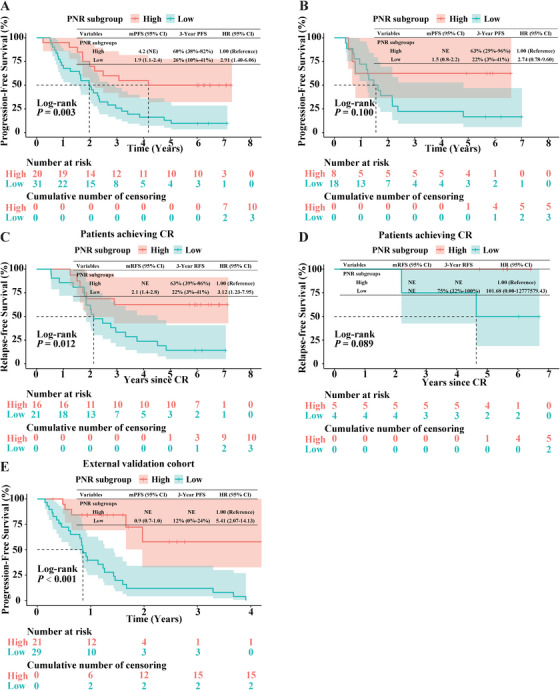
Prognostic value of PNR in R/R cHL with different anti‐PD‐1 regimens. (A and B) Kaplan–Meier survival curves of progression‐free survival (PFS) in patients with high and low PNR levels after treated with decitabine‐plus‐camrelizumab combination (A) or camrelizumab monotherapy (B). (C and D) Kaplan–Meier survival curves of relapse‐free survival (RFS) in CR patients with high and low PNR levels after treated with decitabine‐plus‐camrelizumab combination (C) or camrelizumab monotherapy (D). The median PFS and RFS (95% CI) are shown. Plus signs indicate censored data. (E) Kaplan–Meier survival curves of progression‐free survival (PFS) in patients with high and low PNR levels after treated with anti‐PD‐1‐based therapy in patients with R/R cHL in the external validation cohort.

We next examined whether the prognostic value of PNR was correlated with certain anti‐PD‐1 regimen. First, we analyzed the patients baseline characteristics within these two subgroups, and no statistically significant clinicopathological variables were observed (Table S3). As illustrated in Figure 3A, cases with high PNR had notably longer PFS as compared with those with low PNR after receiving decitabine‐plus‐camrelizumab therapy (median PFS, 4.2 years vs. 1.9 years, *p* = 0.003). Similarly, the superiority in RFS was detected in PNR^high^ versus PNR^low^ cases after decitabine‐plus‐camrelizumab therapy (*p* = 0.012; Figure 3C). Moreover, patients in the PNR^high^ subgroup acquired longer PFS as compared with those in the PNR^low^ subgroup following camrelizumab monotherapy, although statistical significance was not reached (median PFS, not evaluated vs. 1.5 years, *p* = 0.1; Figure 3B). Because of the limited number of patients who achieved CR with camrelizumab monotherapy (*n* = 9), significance was not obtained between these two subgroups (*p* = 0.089; Figure 3D).

Finally, to validate the reliability of PNR cutoff value of 51.6 and mitigate any bias due to the limited sample size, we further studied in an external cohort with R/R cHL after receiving immunotherapy. Patients were stratified into PNR^high^ and PNR^low^ subgroups using the cutoff value of 51.6, the KM analysis substantiated the prognostic prediction value of PNR and reinforced the conclusion that patients with higher pretreatment PNR level were more prone to benefit from anti‐PD‐1‐based immunotherapy (*p* < 0.001; Figure 3E).

### 2.6 Association of PNR and Survival After anti‐PD‐1 Combination Therapy in Solid Tumor Patients

Furthermore, we intended to assess whether PNR could predict the prognosis of anti‐PD‐1‐containing therapy in patients with advanced solid tumor, such as gastric carcinoma, colon cancer, biliary tract carcinoma, or melanoma. Using data from our previous studies, we calculated the value of PNR and plotted ROC curves to determine the optimal cut‐off values. The cut‐off value of PNR in advanced gastric carcinoma (34.7) was determined as death within 6 months after anti‐PD‐1 combination therapy, and PNR cut‐off values in advanced biliary tract carcinoma (40.5), colon cancer (56.1), and melanoma (51.8) were determined as tumor progression within 3 months, 6 months, and 4.5 years following immunotherapy, respectively (Figure S3).

**FIGURE 4:**
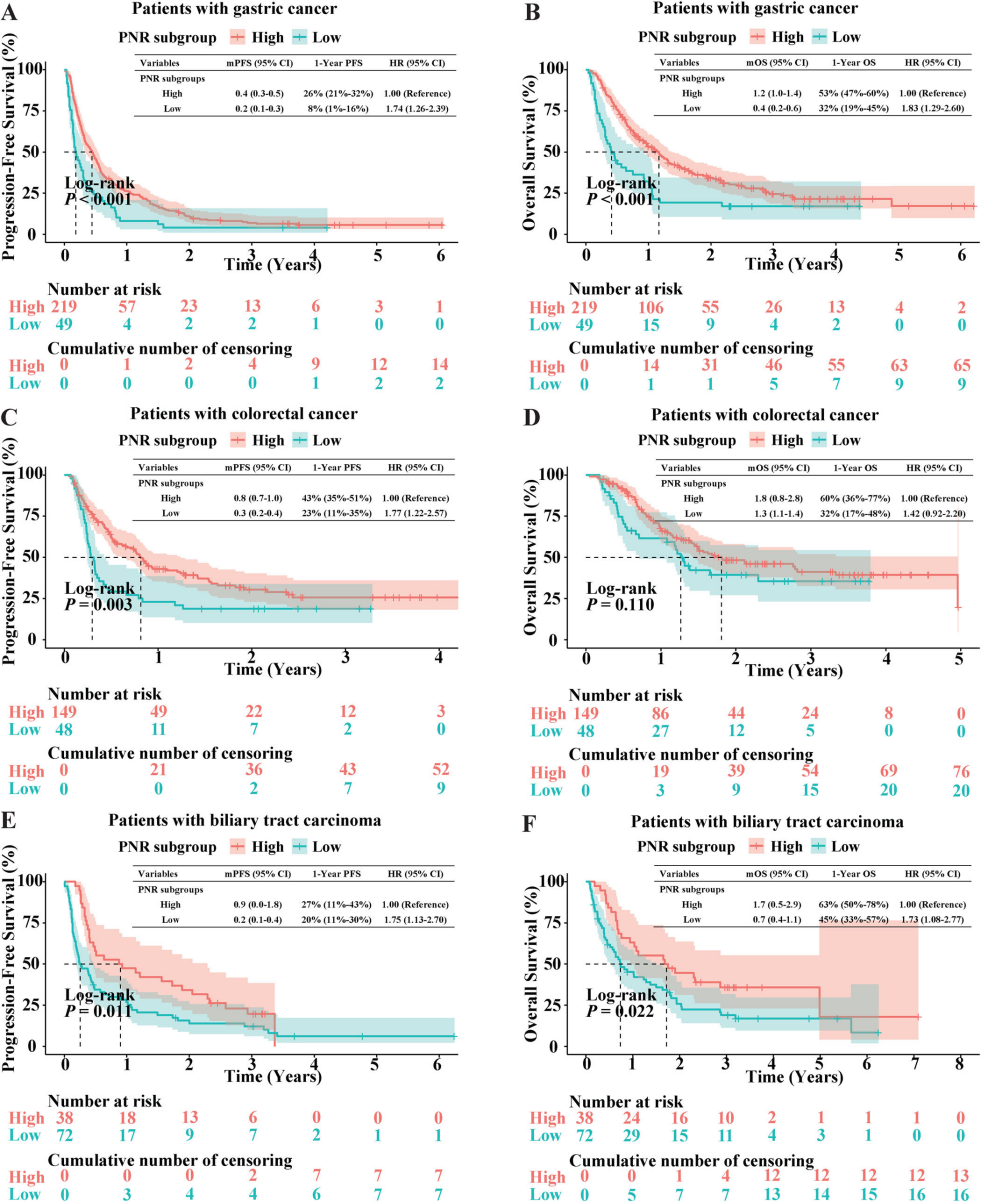
Prognostic value of PNR in patients with advanced solid tumor after receiving anti‐PD‐1 combination therapy. (A, B) Kaplan–Meier survival curves of PFS (A) or overall survival (OS) (B) in advanced gastric carcinoma patients with high PNR (≥ 34.7) and low PNR (< 34.7). (C and D) Kaplan–Meier survival curves of PFS (C) or OS (D) in advanced colon cancer patients with high PNR (≥ 56.1) and low PNR (< 56.1). (E and F) Kaplan–Meier survival curves of PFS (E) or OS (F) in advanced biliary tract patients with high PNR (≥ 40.5) and low PNR (< 40.5). The median PFS and OS (95% CI) are shown.

The results showed that patients with advanced gastric carcinoma, colon cancer, biliary tract carcinoma, or melanoma in the PNR^high^ subgroup all acquired markedly longer PFS as compared with those in the corresponding PNR^low^ subgroup (*p* < 0.001, 0.003, 0.011, and 0.0016, respectively; Figure 4A,C,E and Figure S4). Moreover, patients with advanced gastric carcinoma or biliary tract carcinoma in the PNR^high^ subgroup obtained improved overall survival (OS) versus those in the PNR^low^ subgroup (*p* < 0.001 and 0.022, respectively; Figure 4B,F). Although statistical significance was not detected, advanced colon cancer patients with high pretreatment PNR level acquired longer OS as compared with those with low PNR level (*p* = 0.11, Figure 4D).

## 3 Discussion

Anti‐PD‐1 monotherapy is effective in cases with R/R cHL, and other anti‐PD‐1 combination regimens are widely explored, while potential biomarkers for the clinical efficacy remains limited. In this study, we show that higher pretreatment PNR is associated with increased CR rate and longer PFS after anti‐PD‐1‐based immunotherapy in R/R cHL, and further highlighted the prognostic value of the novel indicator—PNR—in cases with solid tumor after anti‐PD‐1 combination therapy.

In recent years, a large amount of research has focused on applying blood cell composite index as a predictor of the efficacy and long‐term survival of cancer patients treated with immunotherapy. A meta‐analysis showed that high pretreatment NLR and PLR were associated with worse PFS and OS in patients with metastatic renal cell cancer receiving immune checkpoint inhibitors (ICIs) [17, 18, 19, 20, 21, 22, 23]. Similarly, NLR and PLR were reported as poor prognostic indicators in patients with stage III/IV non‐small cell lung cancer or hepatocellular carcinoma with PD‐1 inhibitor [17, 18, 19, 20, 21, 22, 23]. Besides in solid tumors, Tao Y et al. found that PLR had a correlation with prognosis in patients with newly diagnosed early‐stage cHL after chemotherapy or chemo‐radiotherapy, and a new model composed of International Prognostic Score (IPS)‐3 and PLR (IPSPLR) was able to predict freedom from progression for newly diagnosed advanced cHL [17, 18, 19, 20, 21, 22, 23]. This finding suggests that these inflammatory markers may play a role in disease progression and help to identify patients at higher risk for adverse outcomes. However, whether the peripheral blood composite is related to the effectiveness of patients with R/R cHL undergoing immunotherapy remains unclear.

Platelet was regarded as an important guard against viral and bacterial infection, and platelet‐derived biomarkers were demonstrated to play a role in the diagnosis of early serious bacterial infection and sepsis [24]. It has been reported that higher neutrophil count and lower PNR were associated with increased risk of fatal. A study in ovarian cancer patients suggested that the preoperative PNR (≥ 49.2) had a strong association with worse RFS. However, correlations between PNR level and the efficacy and prognosis of immunotherapy in cancer patients has not yet been elucidated. In the present study, to our knowledge, we revealed for the first time the prognostic value of PNR in cHL patients following anti‐PD‐1‐based treatment, which was a superior indicator compared with PLR, NLR, and LMR. Pretreatment PNR (>51.6) positively correlated with higher CR rate and longer PFS after immunotherapy. Interestingly, PNR also served as a prognostic indicator following anti‐PD‐1 combination therapy in solid tumor patients.

The exact mechanism of the correlation between peripheral blood indexes and prognosis of cancer patients has not yet been clarified. Some claimed that it may be related to the tumor microenvironment. Neutrophils took a critical part in the innate immunity, while tumor‐associated neutrophils (TAN) could promote tumorigenesis, metastasis, and cause treatment resistance [25]. The number of TAN in tumor patients was closely related to the resistance to immunotherapy and poor prognosis [25]. PD‐L1^+^ TAN inhibited interferon γ production by CD8^+^ T cells, suppressing the function of cytotoxic T cells [26]. Therefore, higher TAN might be related to worse efficacy of immunotherapy. Platelets account for the largest proportion of circulating cells with immunomodulatory activity, the functions of platelets are more diverse and can also participate in both innate and adaptive immunity [27]. Platelets contributed to CD8 T‐cell priming and provoked an efficient immune response against Listeria challenge [28]. Moreover, platelets regulated various tumor‐associated immune cells and platelet‐leukocyte interaction could initiate and accelerate the inflammatory and immune response [29]. Therefore, the ratio of platelet to neutrophil would be a reasonable and promising biomarker for immunotherapy in tumor patients, offering valuable information for clinical decision‐making.

Overall, PNR stands out as a reliable biomarker to forecast the effectiveness of immunotherapy in multiple advanced tumors. By carefully selecting patients based on the pretreatment PNR level, we can design the antitumor strategies more precisely, thereby reducing unnecessary side effects and ultimately improving patient outcomes.

There were several limitations in this study. (1) Due to the retrospective study design and limited sample size, the accuracy of the results may be affected by retrospective bias such as selection, recall, and measurement. (2) The number of patients treated with anti‐PD‐1 monotherapy was relatively small. (3) Among patients with advanced solid tumor, most were treated with chemotherapy, which would be an important confounding factor. (4) This study was limited to peripheral blood, and further assessment of the abundance and activity of immune cells in the tumor microenvironment was necessary, to explore the mechanisms and functional indicators of immunotherapy.

## 4 Materials and Methods

### 4.1 Research Subjects

This retrospective cohort study included cases with R/R cHL who were either enrolled in our clinical trials (NCT02961101 and NCT03250962) or sourced from the real‐world evidence database at Chinese PLA General Hospital from January 2017 to July 2019. Eligibility criteria included histologically confirmed R/R cHL, age ≥ 12 years, ECOG performance‐status of 0 or 1, with adequate hematological, hepatic, and renal function, and without immunotherapy before. Exclusion criteria included transplantation history, recent ASCT (≤ 100 days), cytotoxic/antibody therapies ≤ 4 weeks pre‐enrollment, active autoimmune/infectious conditions, or life‐threatening comorbidities. The ethics approval (S2016‐127‐01) by Institutional Review Board of Chinese PLA General Hospital was obtained.

Patients with advanced gastric cancer (biliary tract cancer, colon cancer, and melanoma) who came from real world data at the same hospital from December 2014 and May 2021 (September 2015 to April 2021, March 2018 to April 2024, and September 2016 to September 2024) were selected for this study. All patients with solid tumor were 90 years or younger, with ECOG performance‐status of 0 or 1, without conditions potentially affecting peripheral blood albumin and neutrophil counts, such as hematological disorders, infections, viral hepatitis, or cirrhosis, and received at least two cycles of immunotherapy and underwent imaging evaluation. Three ethics approvals (S2021‐642‐01, S2020‐530‐02, and S2019‐136‐01) and solid tumor patients consent were obtained by the same hospital.

### 4.2 Treatment

Among the 77 R/R cHL cases, 26 received camrelizumab monotherapy via intravenous (200 mg), and 51 received decitabine (days 1–5) plus camrelizumab (day 8), per three weeks. An external cohort (*n* = 50) included 11 on decitabine plus anti‐PD‐1 and 39 on chemotherapy plus anti‐PD‐1 regimens. Tumor response was evaluated via ultrasound/CT every two cycles and PET/CT every four cycles (Lugano 2014 criteria). Treatment cessation was permitted after 12‐month CR. Efficacy endpoints included PFS and RFS.

Of the 110 advanced biliary tract cancer patients, 268 advanced gastric cancer patients, 197 advanced colon cancer patients, and 31 advanced melanoma patients, four treatment regimens were included: anti‐PD‐1 monotherapy, anti‐PD‐1 plus anti‐angiogenic therapy, anti‐PD‐1 plus chemotherapy, and triplet regimen consisting ICI, chemotherapy and anti‐angiogenic therapy. Anti‐PD‐1 utilized in this study were outlined as follows: (1) sintilimab was delivered via intravenous infusion at a dosage of 200 mg per 3 weeks; (2) toripalimab was delivered via intravenous infusion at a dosage of 240 mg per 3 weeks; (3) pembrolizumab was calculated based on body weight (3 mg/kg) and administrated per 3 weeks; and (4) nivolumab utilized a biweekly regimen with dose adjustment according to patient weight (2 mg/kg), administered through intravenous infusion. Nivolumab response was first assessed following the third infusion cycle, corresponding to weeks 2–4 post‐treatment initiation. Response assessment for toripalimab, sintilimab, and pembrolizumab was performed after the second treatment cycle, falling within weeks 3–5 of therapy commencement. Clinical response in solid tumor patients was assessed by the change of total length and diameter of all measurable lesions, according to the RECIST1.1 criteria.

### 4.3 Clinical Information Collection and Evaluation

Clinical characteristics of all cases encompassed age, sex, histology, number of lines of prior therapy, immunotherapy regimen, platelet (PLT), absolute neutrophil count (ANC), absolute monocyte count (AMC), and absolute lymphocyte count (ALC). According to the blood routine examination and blood biochemistry, peripheral blood composite indexes of NLR (ANC/ALC), PNR (PLT/ANC), PLR (PLT/ALC), and LMR (ALC/AMC) were calculated. Hematologic biomarkers were quantitatively assessed during the protocol‐defined baseline window (within 7 days preceding immunotherapy initiation).

### 4.4 Statistical Analysis

PFS indicated the period commencing from first dosing to progression or death, and RFS denoted the period commencing from first recorded CR to recurrence or death. Cases without progression or recurrence at database lock date for final analysis or lost to follow‐up were censored.

Data processing and analysis were implemented in R 4.3.0/SPSS 26.0. The continuous variables obtained in this study were statistically analyzed using the median and standard deviation. Meanwhile, all categorical variables were presented as percentages. Survival curves were primarily plotted using Kaplan–Meier and assessed by log‐rank test for comparisons between PNR^high^ and PNR^low^ groups. The PNR threshold was determined by the ROC analysis. Univariate analysis was applied via cox proportional hazards regression and all comparative analyses adopted two‐tailed testing protocols. A *p* value < 0.05 indicated significant difference between the two groups of data.

## Author Contributions

Y.P. collected and analyzed the data, and wrote the paper. X.Z. collected and analyzed the data. C.W., Y.C., X.Q., and N.L. collected the data. Y.L. and W.H. guided the research. J.N. designed the study, analyzed the data, and wrote the paper. All authors have read and approved the final manuscript.

## Ethics Statement

The study was approved by the Ethics Committee of Chinese PLA General Hospital (S2016‐127‐01, S2019‐136‐01, S2020‐530‐02, and S2021‐642‐01) and registered in ClinicalTrials.gov (NCT02961101 and NCT03250962), in accordance with the ethical principles of Good Clinical Practice and the Declaration of Helsinki. Written informed consent was obtained from all participants.

## Conflicts of Interest

All authors declare no conflicts of interest.

## Supporting information



Supporting Information

## Data Availability

The data supporting the findings of this study are available from the corresponding author upon reasonable request.
