# Supplementary material for: Platelet‐to‐Neutrophil Ratio: A Novel Prognostic Indicator for Anti‐PD‐1‐Based Therapy in Relapsed/Refractory Hodgkin Lymphoma and Solid Tumors
Source: MedComm (2020). 2025 May 16;6(6):e70199. doi: 10.1002/mco2.70199 (PMC12082035; doi:10.1002/mco2.70199)
Supplement: Supplementary file 1 — Supporting Information [file MCO2-6-e70199-s001.docx]

**Supplemental data**

**Platelet-to-neutrophil ratio: a novel prognostic indicator for anti-PD-1-based therapy in relapsed/refractory Hodgkin Lymphoma and solid tumors**

Yuting Pan, Xin Zhang, [Chunmeng Wang](https://lib.plagh.cn/s/gov/nih/nlm/ncbi/pubmed/G.https/?term=Wang C[Author]), Nannan Lu, Yang Liu, Yixin Chang, Xueting Qin, Weidong Han and Jing Nie

**Contents**

**[Supplemental Figures](#_Toc2747)** [3](#_Toc2747)

[Figure S1. ROC curves for different peripheral markers. 3](#_Toc25408)

[Figure S2. Prognostic value of PNR in R/R cHL patients with different lines of previous treatment. 4](#_Toc10307)

[Figure S3. ROC of PNR in solid tumor patients. 5](#_Toc22155)

[Figure S4. Kaplan-Meier survival curves of progression-free survival (PFS) in advanced melanoma patients with high and low PNR levels. 6](#_Toc11961)

**[Supplemental Tables](#_Toc20210)** [7](#_Toc20210)

[Table S1. Clinicopathological variables of patients with R/R cHL in the external validation cohort. 7](#_Toc4591)

[Table S2. Association of PNR and the best clinical response of anti-PD-1-base therapy in patients with R/R cHL in the external validation set. 8](#_Toc15208)

[Table S3. Clinicopathological variables within immunotherapy regimen subgroups in patients with R/R cHL. 9](#_Toc6113)

[Table S4. Clinicopathological variables of patients with advanced biliary tract cancer. 10](#_Toc10039)

[Table S5. Clinicopathological variables of patients with advanced gastric carcinoma. 11](#_Toc15524)

[Table S6. Clinicopathological variables of patients with advanced colon cancer. 12](#_Toc23811)

[Table S7. Clinicopathological variables of patients with advanced melanoma. 13](#_Toc6435)

# Supplemental Figures

# Figure S1. ROC curves for different peripheral markers.

#
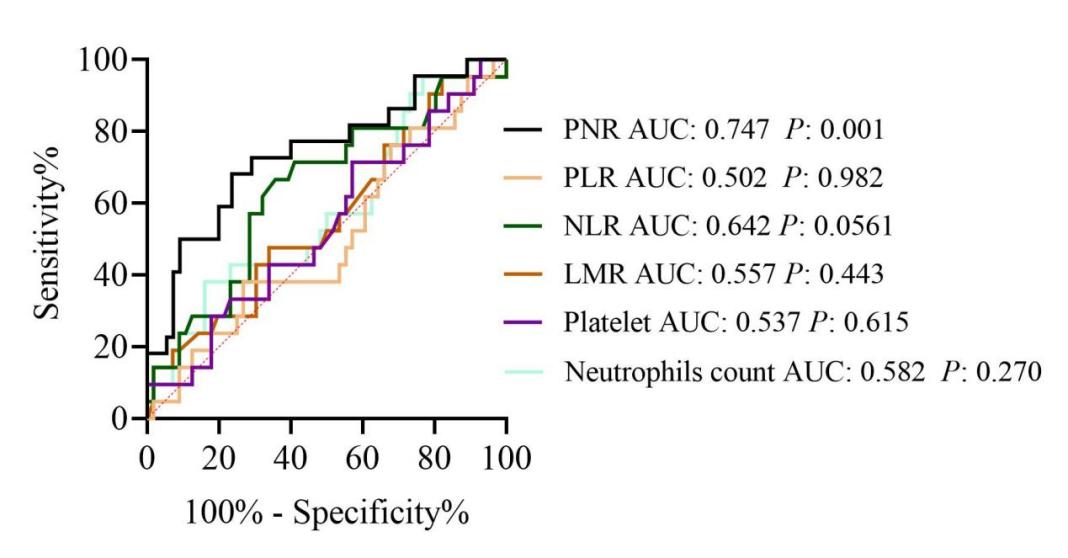


ROC curves of peripheral immune indicators in patients with R/R cHL to assess 6-year tumor progression after anti-PD-1-based therapy. ROC, receiver operator characteristic; LMR, lymphocyte-to-monocyte ratio; PLR, platelet-to-lymphocyte ratio; NLR, neutrophil-to-lymphocyte ratio; PNR, platelet-to-neutrophil ratio; AUC, area under curve.

# **Figure S2. Prognostic value of PNR in R/R cHL patients with different lines of previous treatment.**


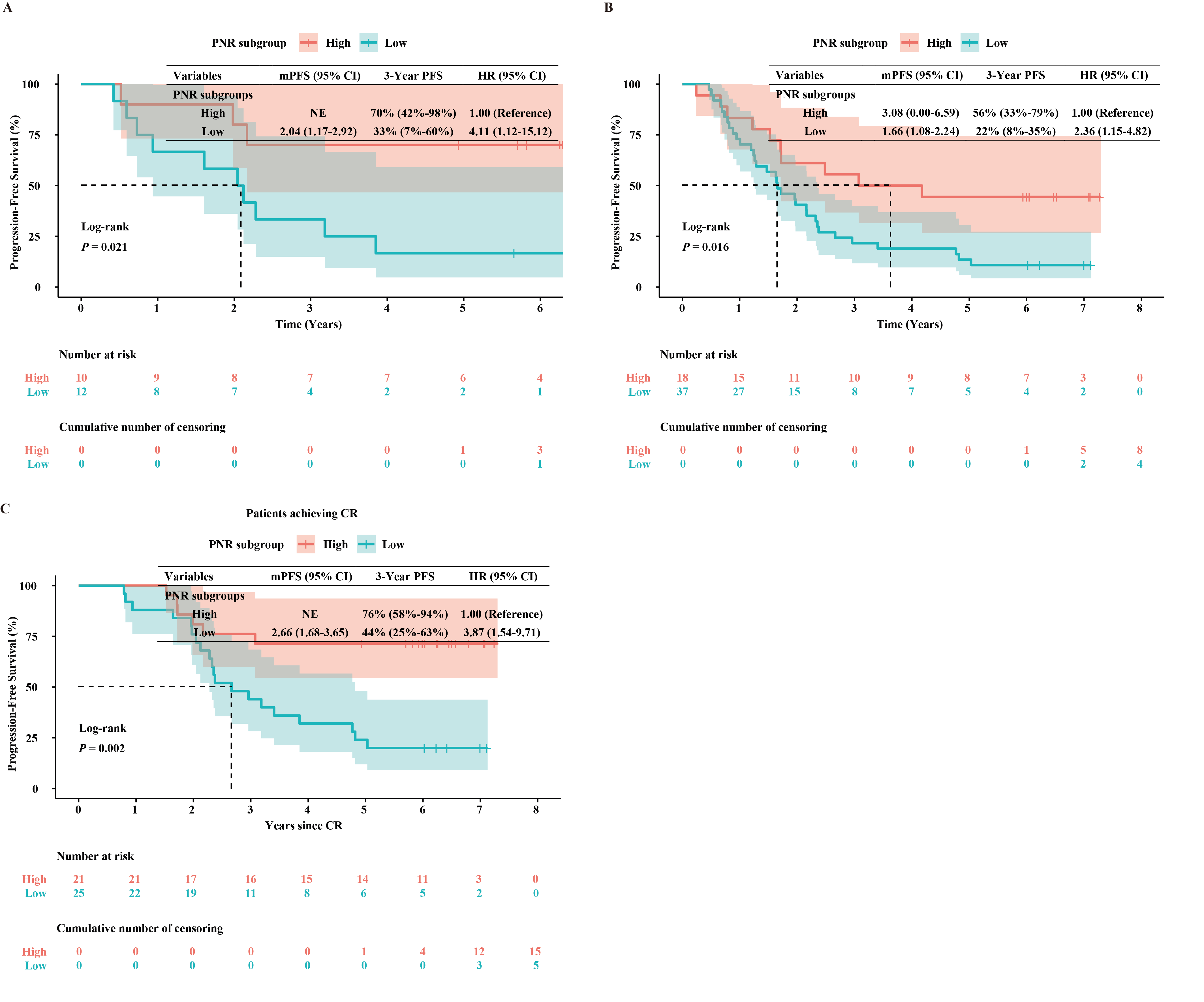


(A, B) Kaplan-Meier survival curves of progression-free survival (PFS) in R/R cHL patients with < 3 (A) or ≥ 3 (B) lines of previous therapy, with high and low PNR levels after anti-PD-1-based therapy. (C) Kaplan-Meier survival curves of PFS in patients achieving CR with high and low PNR levels. The median PFS (95% CI) is shown. Plus signs indicate censored data.

# **Figure S3. ROC of PNR in solid tumor patients.**

# **
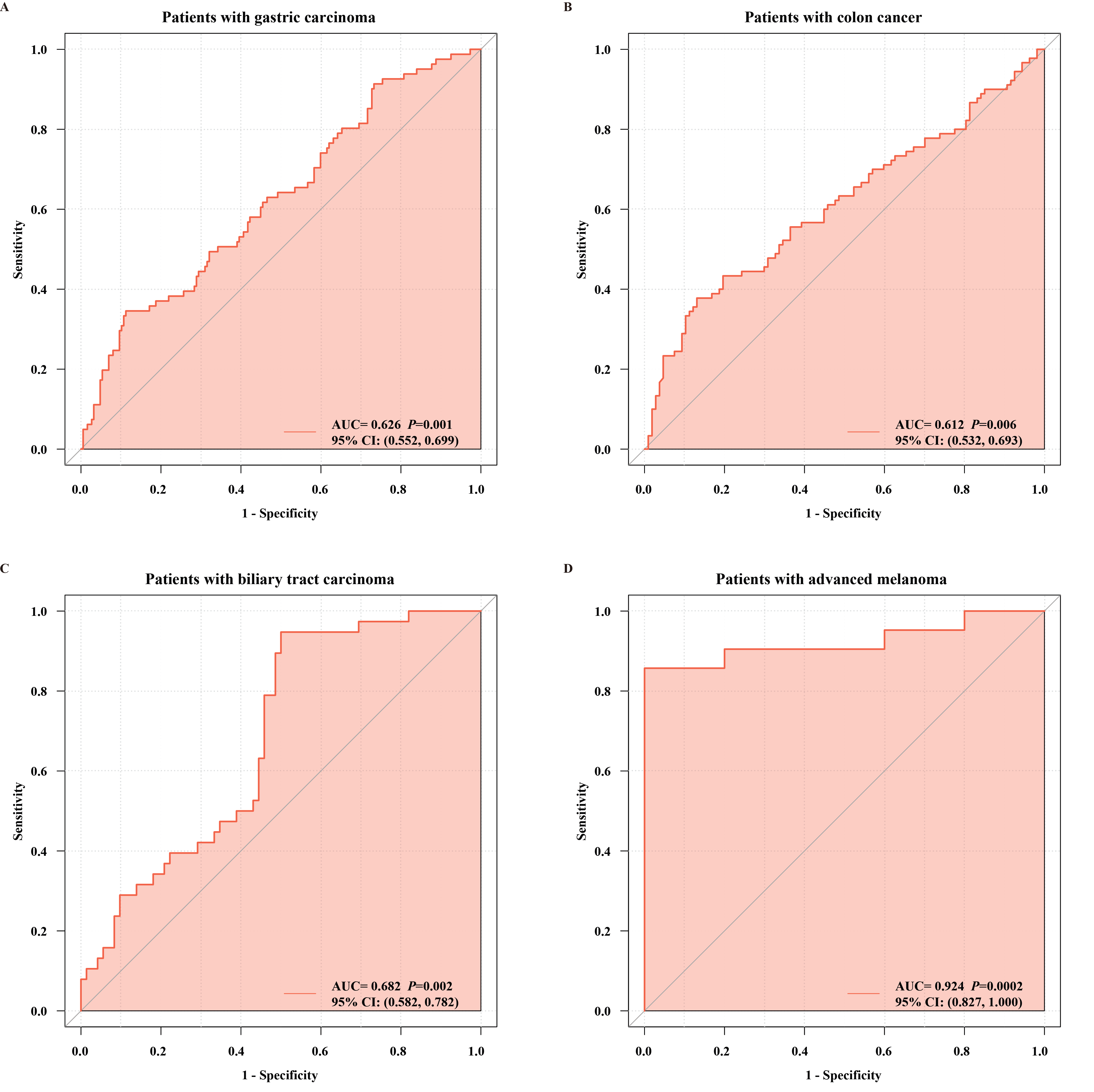
**

1. ROC curves of pretreatment PNR level to assess death at 6 months in patients with advanced gastric cancer after anti-PD-1 combination therapy. (B) ROC curves of pretreatment PNR level to assess tumor progression at 6 months in patients with advanced colon cancer after anti-PD-1 combination therapy. (C) ROC curves of pretreatment PNR level to assess tumor progression at 3 months in patients with advanced biliary tract cancer patients after anti-PD-1 combination therapy. (D) ROC curves of pretreatment PNR level to assess tumor progression at 4.5 years in patients with advanced melanoma after anti-PD-1 combination therapy.

# **Figure S4. Kaplan-Meier survival curves of progression-free survival (PFS) in advanced melanoma patients with high and low PNR levels.**

#
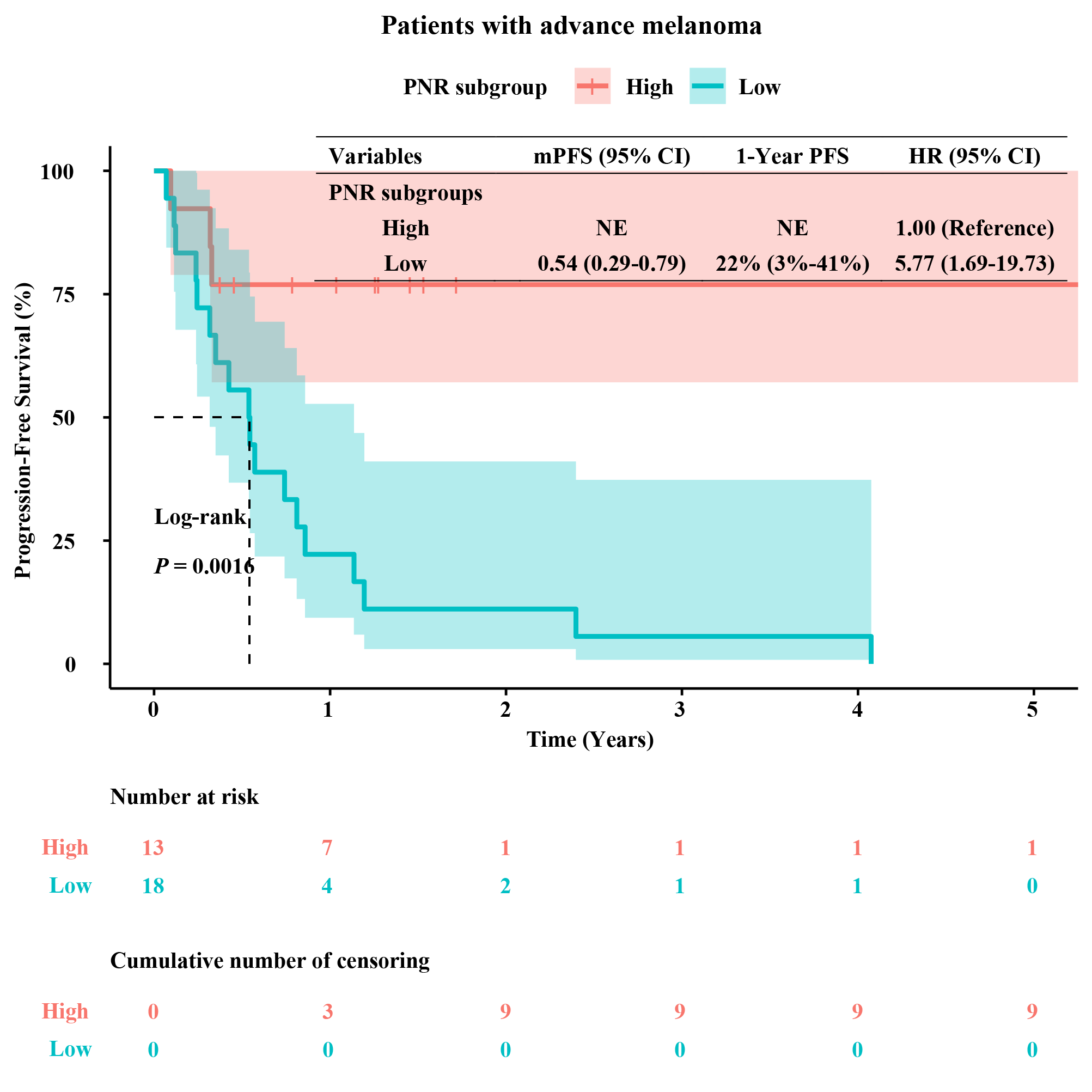


# Kaplan-Meier survival curves of progression-free survival (PFS) in patients with advanced melanoma, with high and low PNR levels after anti-PD-1-based therapy. The median PFS (95% CI) is shown. Plus signs indicate censored data.

# Supplemental Tables

# Table S1. Clinicopathological variables of patients with R/R cHL in the external validation cohort.

| **Characteristics** | **Total**  **(n = 50)** | **PNR^high^ subgroup**  **(n = 21)** | **PNR^low^ subgroup**  **(n = 29)** | ***P* value** |
| --- | --- | --- | --- | --- |
| Age |  |  |  | 0.094 |
| < 29 | 24 (48) | 13 (62) | 11 (38) |  |
| ≥ 29 | 26 (52) | 8 (38) | 18 (62) |  |
| Sex |  |  |  | 0.493 |
| Female | 21 (42) | 10 (52) | 11 (38) |  |
| Male | 29 (58) | 11 (48) | 18 (62) |  |
| Stage |  |  |  | 0.863 |
| II-III | 16 (32) | 7 (33) | 9 (31) |  |
| IV | 34 (68) | 14 (67) | 20 (69) |  |
| Lines of prior therapy |  |  |  | 0.704 |
| < 3 | 27 (54) | 12 (57) | 15 (52) |  |
| ≥ 3 | 23 (46) | 9 (43) | 14 (48) |  |
| Immunotherapy regimen |  |  |  | 0.262 |
| Chemotherapy-plus-anti-PD-1 | 39 (78) | 18 (86) | 21 (72) |  |
| Decitabine-plus-anti-PD-1 | 11 (22) | 3 (14) | 8 (28) |  |

Note: Data are presented as no. (%). A *P* < 0.05 is considered to indicate statistical significance. The *P* values are calculated in SPSS 26.0 using χ^2^ test.

PNR: platelet-to-neutrophil ratio.

# Table S2. Association of PNR and the best clinical response of anti-PD-1-base therapy in patients with R/R cHL in the external validation set.

| **Best clinical response** | **Overall** | **PNR^high^ subgroup** | **PNR^low^ subgroup** | ***P* value** |
| --- | --- | --- | --- | --- |
| **Objective response rate** | 41 (82) | 19 (91) | 22 (76) | 0.184 |
| **CR rate** | 18 (36) | 12 (57) | 6 (21) | 0.008 |

Note: Data are presented as no. (%). A *P* < 0.05 is considered to indicate statistical significance. The *P* values are calculated in SPSS26.0 using χ^2^ test.

# Table S3. Clinicopathological variables within immunotherapy regimen subgroups in patients with R/R cHL.

| **Characteristics** | **Immunotherapy regimen** | | ***P* value** |
| --- | --- | --- | --- |
|  | **Camrelizumab monotherapy** | **Decitabine-plus-camrelizumab** |  |
| Age |  |  | 0.229 |
| < 27 | 15 | 29 |  |
| ≥ 27 | 11 | 22 |  |
| Sex |  |  | 0.694 |
| Female | 10 | 22 |  |
| Male | 16 | 29 |  |
| Histology |  |  |  |
| NSHL | 14 | 34 |  |
| Non-NSHL | 12 | 17 |  |
| Stage |  |  | 0.818 |
| II-III | 10 | 21 |  |
| IV | 16 | 30 |  |
| Previous ASCT |  |  | 0.892 |
| Yes | 7 | 13 |  |
| No | 19 | 38 |  |
| Lines of prior therapy |  |  | 0.819 |
| < 3 | 15 | 30 |  |
| ≥ 3 | 11 | 21 |  |

Abbreviations: NSHL, nodular sclerosis Hodgkin lymphoma; ASCT, autologous stem cell transplant. The *P* values are calculated in SPSS 26.0 using χ^2^ test.

# Table S4. Clinicopathological variables of patients with advanced biliary tract cancer.

| **Characteristics** | **Total**  **(n =268)** | **PNR^high^ subgroup**  **(n = 219)** | **PNR^low^ subgroup**  **(n = 49)** | ***P* value** |
| --- | --- | --- | --- | --- |
| Age |  |  |  | 0.853 |
| < 59 | 129 (48) | 106 (48) | 23 (47) |  |
| ≥ 59 | 139· (52) | 113 (52) | 26 (53) |  |
| Sex |  |  |  | 0.824 |
| Female | 69 (26) | 57 (26) | 12 (25) |  |
| Male | 199 (74) | 162 (74) | 37 (76) |  |
| Histology |  |  |  | 0.240 |
| Cardia | 73 (27.2) | 60 (27) | 13 (27) |  |
| Body/Fundus | 110 (41) | 85 (39) | 25 (51) |  |
| Pylorus | 83 (31) | 72 (33) | 11 (22) |  |
| Miss | 2 (1) | 2 (1) | 0 (0) |  |
| HER-2 |  |  |  | 0.510 |
| Present | 34 (13) | 26 (12) | 8 (67) |  |
| Absent | 177 (66) | 144 (66) | 33 (16) |  |
| Miss | 57 (21) | 49 (22) | 8 (16) |  |
| Lines of prior therapy |  |  |  | 0.222 |
| < 2 | 125 (47) | 106 (48) | 19 (39) |  |
| ≥ 2 | 143 (53) | 113 (52) | 30 (61) |  |
| Anti-PD-1 plus other therapy |  |  |  | 0.319 |
| Yes | 216 (81) | 179 (82) | 37 (76) |  |
| No | 52 (19) | 40 (18) | 12 (25) |  |

Note: Data are presented as no. (%). A *P* < 0.05 is considered to indicate statistical significance. The *P* values are calculated in SPSS 26.0 using χ2 test.

# Table S5. Clinicopathological variables of patients with advanced gastric carcinoma.

| **Characteristics** | **Total**  **(n = 197)** | **PNR^high^ subgroup**  **(n = 149)** | **PNR^low^ subgroup**  **(n = 48)** | ***P* value** |
| --- | --- | --- | --- | --- |
| Age |  |  |  | 0.052 |
| < 54 | 97 (49) | 78 (48) | 19 (40) |  |
| ≥ 54 | 100 (51) | 71 (52) | 29 (60) |  |
| Sex |  |  |  | 0.062 |
| Female | 67 (34) | 56 (38) | 11 (23) |  |
| Male | 130 (66) | 93 (62) | 37 (71) |  |
| KRAS |  |  |  | 0.662 |
| Absent | 92 (47) | 69 (47) | 23 (48) |  |
| Present | 75 (38) | 54 (37) | 21 (44) |  |
| Miss | 30 (15) | 25 (17) | 4 (8) |  |
| BRAF |  |  |  | 0.350 |
| Absent | 154 (78) | 112 (75) | 42 (88) |  |
| Present | 13 (7) | 11 (7) | 2 (4) |  |
| Miss | 30 (15) | 26 (17) | 4 (8) |  |
| HER-2 |  |  |  | 0.135 |
| Absent | 106 (54) | 83 (56) | 23 (48) |  |
| Present | 35 (18) | 23 (15) | 12 (25) |  |
| Miss | 56 (28) | 43 (29) | 13 (27) |  |
| Lines of prior therapy |  |  |  | 0.663 |
| < 2 | 75 (38) | 58 (39) | 17 (35) |  |
| ≥ 2 | 122 (62) | 91 (61) | 31 (65) |  |
| Anti-PD-1 plus other therapy |  |  |  | 0.386 |
| Yes | 46 (23) | 37 (25) | 9 (19) |  |
| No | 151 (77) | 112 (75) | 39 (81) |  |

Note: Data are presented as no. (%). A *P* < 0.05 is considered to indicate statistical significance. The *P* values are calculated in SPSS 26.0 using χ2 test.

# Table S6. Clinicopathological variables of patients with advanced colon cancer.

| **Characteristics** | **Total**  **(n = 110)** | **PNR^high^ subgroup**  **(n = 38)** | **PNR^low^ subgroup**  **(n = 72)** | ***P* value** |
| --- | --- | --- | --- | --- |
| Age |  |  |  | 0.589 |
| < 60 | 57 (52) | 20 (53) | 37 (51) |  |
| ≥ 60 | 53 (48) | 18 (47) | 35 (49) |  |
| Sex |  |  |  | 0.638 |
| Female | 43 (39) | 16 (42) | 27 (38) |  |
| Male | 67 (61) | 22 (58) | 45 (63) |  |
| Histology |  |  |  | 0.423 |
| Bile ducts | 88 (80) | 32 (84) | 56 (78) |  |
| Gall bladder | 22 (20) | 6 (16) | 16 (22) |  |
| Lines of prior therapy |  |  |  | 0.446 |
| < 2 | 79 (72) | 29 (76) | 50 (69) |  |
| ≥ 2 | 31 (28) | 9 (24) | 22 (31) |  |
| Anti-PD-1 plus other therapy |  |  |  | 0.962 |
| Yes | 90 (82) | 31 (82) | 59 (82) |  |
| No | 20 (18) | 7 (18) | 13 (18) |  |

Note: Data are presented as no. (%). A *P* < 0.05 is considered to indicate statistical significance. The *P* values are calculated in SPSS 26.0 using χ2 test.

# Table S7. Clinicopathological variables of patients with advanced melanoma.

| **Characteristics** | **Total**  **(n = 31)** | **PNR^high^ subgroup**  **(n = 13)** | **PNR^low^ subgroup**  **(n = 18)** | ***P* value** |
| --- | --- | --- | --- | --- |
| Age |  |  |  | 0.409 |
| < 59 | 14 (45) | 7 (54) | 7 (49) |  |
| ≥ 59 | 17 (55) | 6 (46) | 11 (51) |  |
| Sex |  |  |  | 0.592 |
| Female | 8 (26) | 4 (31) | 4 (39) |  |
| Male | 23 (74) | 9 (69) | 14 (61) |  |
| Location |  |  |  | 0.171 |
| Skin | 14 (45) | 4 (31) | 10 (56) |  |
| Non-Skin | 17 (55) | 9 (69) | 8 (44) |  |
| Stage |  |  |  | 0.605 |
| II-III | 15 (48) | 7 (54) | 8(44) |  |
| IV | 16 (52) | 6 (46) | 10 (56) |  |
| Lines of prior therapy |  |  |  | 0.291 |
| < 1 | 20 (65) | 7 (54) | 13 (72) |  |
| ≥ 2 | 11 (36) | 6 (46) | 5 (28) |  |
| Immunotherapy regimen |  |  |  | 0.470 |
| anti-PD-1 monotherapy | 12 (39) | 6 (46) | 6 (33) |  |
| anti-PD-1 combination therapy | 19 (61) | 7 (54) | 12 (67) |  |

Note: Data are presented as no. (%). A *P* < 0.05 is considered to indicate statistical significance. The *P* values are calculated in SPSS 26.0 using χ^2^ test.

PNR: platelet-to-neutrophil ratio.
